# Pediatric Burn Treatment with Non-Thermal Atmospheric Plasma and Epifast^®^: Clinical Results

**DOI:** 10.3390/ebj6020020

**Published:** 2025-04-14

**Authors:** Pablo Rodríguez-Ferreyra, Régulo López-Callejas, Teresa Narváez-Robles, Benjamín Gonzalo Rodríguez-Méndez, Omar Israel Gayosso-Cerón, Antonio Mercado-Cabrera, Irene Lule-Reyna, Othoniel Mondragón-Dagio, Raúl Valencia-Alvarado, Jesús Duarte-Mote

**Affiliations:** 1 Pediatric Burn Unit, Dr. Nicolás San Juan General Hospital, Mexico State Health Institute, Toluca 50010, Mexico; pablomx1965@gmail.com (P.R.-F.); terenarvaezr@gmail.com (T.N.-R.); drgayosso@gmail.com (O.I.G.-C.); sirelule@yahoo.com.mx (I.L.-R.); mdo2.surgery@gmail.com (O.M.-D.); 2 Plasma Physics Laboratory, National Institute for Nuclear Research, Ocoyoacac 52750, Mexico; regulo.lopez@inin.gob.mx (R.L.-C.); antonio.mercado@inin.gob.mx (A.M.-C.); raul.valencia@inin.gob.mx (R.V.-A.); 3 Teaching and Research Unit, Dr. Nicolás San Juan General Hospital, Mexico State Health Institute, Toluca 50010, Mexico; jesusdm3@yahoo.com.mx

**Keywords:** superficial partial-thickness burn, deep dermal burn, graft application, non-thermal atmospheric pressure plasma, wounds, healing, biomedical devices

## Abstract

The effective treatment of severe burns in pediatric patients is essential for minimizing complications and promoting optimal recovery. This study investigates the use of non-thermal atmospheric pressure plasma (NTAPP) as an adjuvant therapy in combination with Epifast^®^ for the experimental group, compared to standard care involving early excisions and Epifast^®^ for the control group. A randomized controlled trial was conducted with 40 pediatric patients suffering from superficial partial-thickness and deep dermal burns. The experimental group that received NTAPP daily demonstrated a significant reduction in the need for skin grafts, requiring only 10% compared to 40% in the control group (*p* = 0.02). Although there were no statistically significant differences in the length of hospital stay, the experimental group showed a trend toward shorter stays (9.85 days vs. 11.65 days; *p* = 0.38) and lower analgesic consumption (13.01 doses vs. 21.15 doses; *p* = 0.09). Additionally, the infection rate in the NTAPP-treated group was significantly lower at 25%, compared to 37.95% in the control group (*p* < 0.05). These findings suggest that NTAPP enhances wound healing while reducing surgical morbidity and the risk of infections. In conclusion, this study highlights the transformative potential of NTAPP as an innovative strategy in pediatric burn management. It combines clinical efficacy with a less invasive approach, representing a significant advance in regenerative medicine and opening new avenues for research into advanced therapies.

## 1. Introduction

Pediatric burns represent a significant public health burden, with consequences ranging from morbidity to mortality. These injuries urgently need effective treatments to accelerate healing and prevent complications. Despite advances in burn management, challenges remain in preventing and treating these injuries, underscoring the importance of continued research in this field [1,2,3,4].

Recent advances in the field of regenerative medicine have led to the development of new therapies for the treatment of burns. Non-thermal atmospheric pressure plasmas (NTAPPs) have shown particular promise [5]. The application of NTAPP, which can be performed at room temperature and without the risk of thermal damage, has remarkably accelerated wound healing [6,7]. Whether by direct contact with the tissue or through a dielectric barrier discharge device [8,9], NTAPP may offer a safe and effective therapeutic option for treating burns.

NTAPP has emerged as a promising therapeutic tool in wound management, mainly for burns. The ambient temperature characteristic of these plasmas [9], combined with the generation of high-energy electrons, induces the formation of various reactive oxygen and nitrogen species (RONS), such as hydrogen peroxide (H_2_O_2_), hydroxyl radicals (^•^OH), and nitrogen dioxide (NO_2_). These RONS are instrumental in antimicrobial activity, eliminating pathogens and creating an environment conducive to healing [10,11]. In addition, the NTAPP generates nitric oxide (NO), a signaling molecule crucial in skin physiology, including wound healing, response to stimuli, and vascular regulation [12,13]. NO production by NTAPP can modulate inflammatory processes and promote angiogenesis, facilitating tissue repair.

Preclinical research has been instrumental in understanding the mechanisms of action and the therapeutic potential of NTAPP. Numerous studies, mainly in animal models, have explored their applications in various medical fields [10,14]. The results obtained to date have been promising and laid the groundwork for the transition from preclinical to clinical research to evaluate the efficacy and safety of NTAPPs in treating various pathologies [9,14]. Initial clinical trials have demonstrated the efficacy of NTAPP in diabetic foot ulcers, oral mucosal lesions, deep neck abscesses, anal fistulas, and anastomotic leaks [15,16]. These findings and its mechanism of action suggest that NTAPP may be a promising adjuvant therapy in treating burns. However, further clinical research is required to thoroughly evaluate its potential in this area.

Several factors influence the therapeutic efficacy of NTAPP, including the composition of the gas used, the duration of exposure, the distance between the plasma source and the tissue, and the electrical energy applied [17]. These parameters determine the dose of reactive species, UV radiation, and the generated electric field, which directly influence the biological response of the tissue. Recent studies have shown that helium-generated NTAPP can activate platelets and stimulate fibrin formation, suggesting a key role in hemostasis and tissue repair [6].

The findings and mechanisms of action indicate that NTAPP therapy enhances wound healing through several important processes [18]. It promotes cell regeneration, modulates the inflammatory response, and encourages angiogenesis while protecting surrounding tissues from damage [19]. Additionally, NTAPP possesses sterility properties that help reduce the risk of infection, further improving the healing process [20]. These beneficial effects are linked to its interactions with key cells involved in tissue repair, such as fibroblasts, keratinocytes, and macrophages [21]. However, further research is needed to confirm these findings and determine the clinical efficacy of NTAPP as an adjunct therapy for burn treatment [22]. This makes it particularly valuable for burn patients, where maintaining tissue integrity and minimizing pain are crucial [23], especially in pediatric cases [24,25].

The application of NTAPP in the healing of chronic or surgical wounds could yield results comparable to or even surpassing those of conventional methods, indicating the need for further investigation [6]. Preliminary evidence suggests that NTAPP may reduce the necessity for skin grafts compared to traditional techniques, leading to faster and more effective healing [26,27]. Regarding pain management, NTAPP has demonstrated benefits by creating a moist environment that promotes granulation and may lessen discomfort during treatment [28]. This aspect is vital for enhancing patients’ well-being and overall experience. Additionally, NTAPP has been shown to decrease the number of procedures needed for effective wound management [29], which benefits patients by minimizing the trauma associated with multiple interventions and optimizes resource use in clinical settings.

In this context, advanced technologies like NTAPP have been shown to reduce infection rates. This therapeutic approach can help create a sterile environment and enhance blood flow to the wound, both vital for optimal healing. Recent studies [30,31] indicate that NTAPP helps eliminate pathogenic microorganisms and aids in tissue regeneration, leading to a more efficient healing process.

This study aims to evaluate whether NTAPP improves the treatment of second-degree burns compared to a conventional protocol. Aspects such as healing speed, scar quality, pain, and length of hospital stay will be analyzed to determine whether NTAPP can offer better outcomes in managing these injuries.

## 2. Materials and Methods

### 2.1. Patients

Two different protocols are compared: the first, a conventional approach involving debridement, the application of Epifast^®^, and a dressing; the second, which consists of surgical cleansing for antisepsis, followed by the application of NTAPP directly to the wound, covered with Epifast^®^ and dry gauze, and then the application of NTAPP every 24 h without removing the Epifast^®^.

The project adhered to the standards of the Declaration of Helsinki: ethical principles for medical research involving human beings, as well as the guidelines of the Research and Research Ethics Committee of the “Mónica Pretelini Sáenz” Maternal-Perinatal Hospital, belonging to the Mexico State Health Institute (ISEM). The medical protocol was duly approved, considering this research as higher than minimal risk. All selected patients met the criteria established in the approved medical protocol, with the Federal Commission for the Protection Against Sanitary Risks (COFEPRIS) records 13CI15-106-068 and 15CEI-005-20170615. The patients were selected from those admitted to the Pediatric Burn Unit of the Dr. Nicolás San Juan General Hospital of ISEM, Mexico.

**Figure 1:**
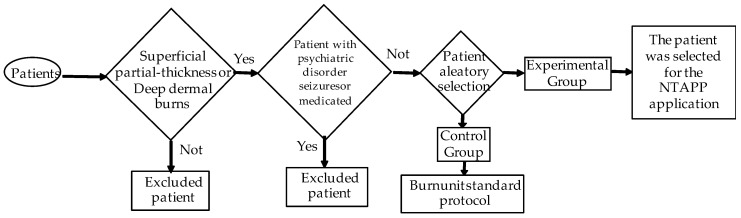
Scheme of the group selection procedure.

The inclusion criteria for this study included patients with both superficial partial-thickness and deep dermal burns (see Figure 1). The burns are classified into superficial partial thickness and deep dermal based on the depth of dermal involvement. Superficial partial-thickness burns affect the epidermis and upper dermis, characterized by redness, swelling, blistering, severe pain, and a moist, shiny surface. Deep dermal burns extend into the deeper dermis, presenting with similar symptoms but also potential nerve damage, resulting in variable pain and a dry, red, or white appearance. Skin grafting may be required for the optimal healing of deep dermal burns. This study focused on patients with second-degree burns, differentiating between superficial partial-thickness and deep dermal burns. At least two experts assessed burn depth using standardized criteria, including skin color, blister presence, and patient-reported pain, aligning with the described classification. In addition, a puncture test with a sterile needle was performed to determine the sensitivity in the affected area. This rigorous assessment was necessary due to limited resources for performing skin biopsies or advanced imaging studies in our unit. Patients ranging from newborns to 15 years of age were included, with no restrictions regarding body mass index or physical status. Informed consent was obtained from the responsible family member, who agreed to the patient’s participation in this study.

Exclusion criteria for this study were carefully defined to ensure the validity and integrity of the results. First, it is essential to note that full-thickness burns, which involve all layers of the skin—epidermis, dermis, and subcutaneous tissue—and can even affect deeper structures such as muscles and bones, are excluded from the study. Furthermore, due to the destruction of nerve endings, pain is not experienced in the affected area. Full-thickness burns require a specialized therapeutic approach, including immediate medical treatment and, in many cases, skin grafts for proper healing, which could significantly interfere with the study objectives. Patients with psychiatric disorders, a history of seizures, and those receiving medications that affect the central nervous system are also excluded, as these conditions could influence the response to treatment. Exclusion criteria also include patients who chose not to participate in this study and those who withdrew their consent or opted not to continue treatment. These criteria have been established to ensure a homogeneous study population and that the results are representative and clinically relevant.

This study included a total of 40 patients who were divided into two groups: a control group and an experimental group that received NTAPP therapy. Debridement was carried out between the fourth and sixth days after the burn. This timeframe is optimal for accurately assessing the depth and extent of the injuries, which is essential for effective treatment planning. Although the risk of infection may rise due to the inflammatory response during this period, it is crucial to avoid premature debridement, which could increase morbidity and complications. Burn wounds were evaluated during this time, and treatment was customized to meet the specific needs of each wound. Before debridement, which was performed under general anesthesia, the burned area was treated with a petroleum jelly dressing and secured with an elastic bandage to protect the wound and promote healing.

Assessing infection rates is crucial to wound management, particularly for burns. Signs of infection at the wound site can include redness, warmth, swelling, pus discharge, and fever. Microbiological testing is essential to confirm a suspected infection, identify potential pathogens, and guide appropriate treatment.

The control group received the standard treatment protocol of the Pediatric Burn Unit of Dr. Nicolás San Juan General Hospital. This protocol includes debridement, and a biological dressing composed of human keratinocyte cultures (Epifast^®^ releases growth factors, including transforming growth factor (TGF)) was applied, covered with dry gauze, and secured with a bandage [32]. In the experimental group, patients underwent surgical cleaning with an antiseptic solution without the debridement of the burn wounds under general anesthesia, followed by the direct application of the NTAPP procedure to the burn site. Following the NTAPP procedure, a biological dressing composed of human keratinocyte cultures (Epifast^®^) was applied, and the treated area was covered with dry gauze and secured with a bandage. The secondary dressing (gauze) was replaced every 24 h after the NTAPP application and then covered with gauze or a dry compress. This procedure was followed until the wound healed.

Our protocol established the standard of care for skin grafting for patients with total body surface area burns exceeding 10% who did not respond adequately to initial treatment.

Effective pain management is critical for pediatric patients with both superficial partial-thickness and deep dermal second-degree burns. Our specialized unit employs a comprehensive approach to ensure optimal pain control throughout the perioperative period. Preoperatively, intravenous Paracetamol (Acetaminophen) is prioritized to reduce pain and alleviate pre-procedural anxiety, facilitating smoother transitions into surgery. During and immediately following the procedure, Buprenorphine is administered to provide robust analgesia, selected for its efficacy and safety in pediatric populations. Continuous pain assessment allows us to tailor the analgesic regimen to individual patient needs, making necessary adjustments to balance pain relief with minimizing adverse effects. In our practice, we implement a systematic antibiotic strategy to prevent secondary bacteremia and reduce infection risks in burn patients. We implement a systematic antibiotic strategy to mitigate the risks of secondary bacteremia and infection in burn patients. Systemic antibiotics such as intravenous dicloxacillin or aztreonam are administered under general anesthesia, immediately before surgical intervention, providing broad-spectrum coverage against potential pathogens. When infection is confirmed through positive cultures, antibiotic regimens are adjusted based on bacterial sensitivity profiles. As part of our protocol, microbiology swabs are taken from each patient during debridement to guide these adjustments. In such cases, vancomycin and ciprofloxacin are utilized as first-line antibiotics, targeting the prevalent pathogens commonly found in burn patients, including *Staphylococcus epidermidis* and *Pseudomonas aeruginosa*.

### 2.2. Instrumentation

**Figure 2:**
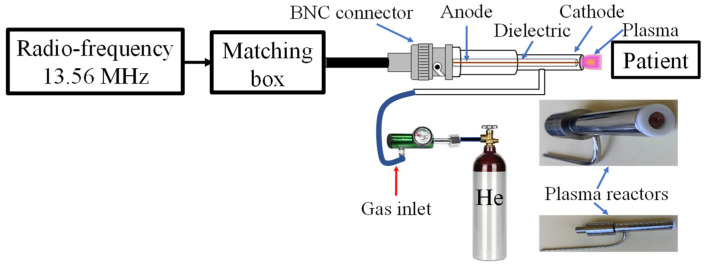
Schematic of the NTAPP generator.

Consistent with our previous studies on NTAPP clinical applications [15,16], we have developed a novel plasma reactor (Figure 2) specifically designed for burn wound treatment. The reactor incorporates an 8 mm outlet nozzle, optimized through simulations and experimentation to achieve a balance between wide coverage and uniform plasma distribution.

Helium, chosen for its high ionization efficiency, inert nature, and excellent safety profile, was utilized as the working gas. A constant flow rate of 0.5 L/min was maintained, while operating the 13.56 MHz radio-frequency source at a power range of 18–20 W. This configuration produced an irradiance ranging from 0.420 to 0.560 W/cm^2^, with an average irradiance of 0.4659 W/cm^2^ applied to the burn wounds. Importantly, this irradiance level is lower than the safety threshold of 4 W/cm^2^ recommended by the International Commission on Non-Ionizing Radiation Protection (ICNIRP) [33], ensuring the safety of the patient’s tissues during treatment. Additionally, we maintained the plasma reactor outlet temperature below 27 °C to preserve tissue integrity and prevent thermal damage. Treatment durations, which were adjusted based on the wound surface area, demonstrate the adaptability of the NTAPP treatment, with an average exposure time of approximately 1 min per 10 cm^2^ [16]. This adaptability ensures that the NTAPP treatment is personalized and effective.

**Figure 3:**
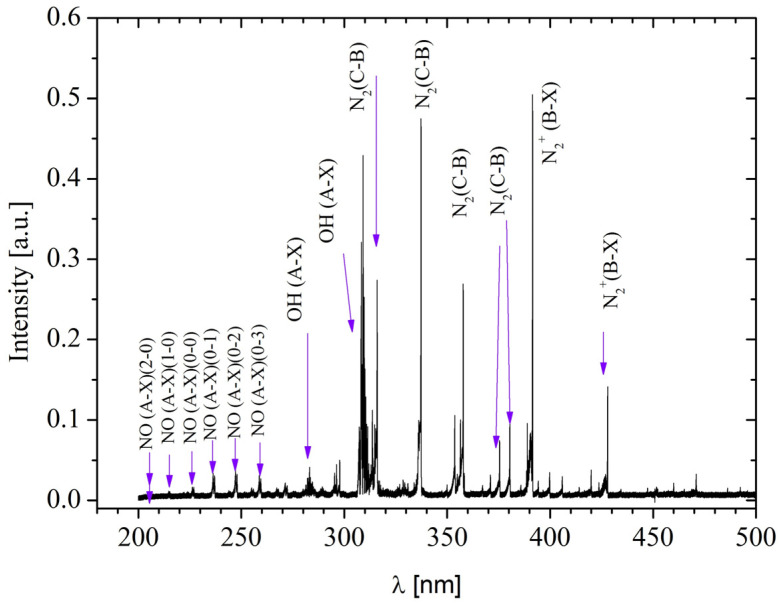
NTAPP spectrum.

The generated plasma comprises a diverse range of reactive species (Figure 3), including reactive oxygen species (ROS) such as ^•^OH, singlet oxygen (O_2_^1^Δg), and H_2_O_2_, determined by absorbance. The reactive species profile was evaluated through multiple measurements, and an average of the reactive species generated for each treatment was determined, which allowed the identification of the dominant reactive species in each case. Among the ROS, H_2_O_2_ stands out for its antibacterial properties and ability to promote healing. ^•^OH is highly reactive and contributes to the destruction of pathogens, while singlet oxygen promotes collagen production. Concurrently, reactive nitrogen species (RNS), including NO in its γ phase, NO_2_, and molecular nitrogen ions (N_2_^+^), were also generated. NO is crucial for vasodilation and the regulation of inflammation, while peroxynitrite (ONOO^−^) may have mixed effects on healing. These reactive oxygen and nitrogen species enable the sterilization of burn wounds while simultaneously promoting wound healing, an effect consistent with observations by other authors [34,35]. Furthermore, NTAPP has demonstrated efficacy in eliminating various types of bacteria [36,37,38].

The plasma reactor was calibrated before each use to ensure accurate and reproducible results. This involved verifying the flow rate of helium gas and the power output of the radio-frequency source, as well as monitoring the temperature of the plasma in the interaction zone. All measurements were conducted using high-precision instruments to maintain consistency across all treatments.

By expanding the reactor nozzle and optimizing the operational parameters, we aimed to achieve a more uniform and practical application of NTAPP over burn wounds. This setup allowed for a controlled and safe plasma delivery, enhancing the overall treatment efficacy while adhering to established safety standards.

### 2.3. Statistical Analysis

We employed a comprehensive statistical analysis using appropriate tools for different data characteristics to evaluate the observed differences. Given the data’s non-normal distribution, the Wilcoxon signed-rank test was utilized to compare medians. This non-parametric test provided a robust method for assessing differences in paired samples. For comparisons between independent groups, Student’s *t*-test was applied, assuming a normal distribution within those groups.

Both tests were evaluated at a significance level of *p* < 0.05, indicating a low probability that the observed differences occurred by chance. This dual approach—using the Wilcoxon test for non-normally distributed data and Student’s *t*-test for normally distributed independent groups—ensured a thorough and reliable statistical assessment. Together, these methods strengthened the validity of our findings, providing a solid statistical foundation for the significance of the observed outcomes.

## 3. Results

Demographic analysis has revealed a significant trend (Table 1): the pediatric population aged 1 to 4 years is the most vulnerable to burns, constituting 62.5% of all cases. While the mean age between the control (4.25 years) and experimental (3.97 years) groups did not significantly differ across the population, further subgroup analysis revealed intriguing patterns. Notably, girls in the experimental group had a significantly older mean age (4.57 years) than their counterparts in the control group (3.23 years). In contrast, boys in the control group had a slightly older mean age (5.18 years) than the experimental group (4.69 years). These findings point to subtle but potentially crucial differences in age and sex distribution between the groups, which could have significant implications for understanding the specific risk factors related to exposure to burn-causing agents in each group and, ultimately, for preventing and treating pediatric burns.

Our analysis of the causes of burns has revealed important insights that could significantly impact the prevention of burn injuries. Scalds were found to be the most frequent cause (72.5%), followed by fire burns (20%) and electrical burns (7.5%). The temporal distribution of these events was also a key issue. A significant concentration of cases was observed between 12:00 and 24:00 h, representing 80% of the total. Burns from the fire peaked during the afternoon (12:00 to 18:00 h), with 15% of cases occurring during this time. Although less frequent, electrical burns peaked in the same interval, highlighting our research to prevent such incidents.

Table 2 presents the results of patients admitted to the Pediatric Burn Unit. The table includes data for the control group and the experimental group. We used the Lund and Browder graph to establish the extent of %total body surface area (TBSA) burned on the patients. The TBSA was calculated by summing the percentages of burned areas across various body regions: head and neck, anterior trunk, posterior trunk, right upper extremity, left upper extremity, right lower extremity, and left lower extremity. Each patient’s TBSA for these regions was assessed individually and then aggregated to determine the TBSA. The comparative analysis of TBSA between the control group and the experimental group showed closely matched values. Specifically, TBSA values for the control group and the experimental group (12.60% vs. 12.55%) were derived from the following average regional contributions: head and neck (1.1% vs. 2.02%), anterior trunk (2.0% vs. 2.5%), posterior trunk (1.68% vs. 1.41%), right upper extremity (1.55% vs. 1.2%), left upper extremity (1.15% vs. 1.09%), right lower extremity (2.8% vs. 1.91%), and left lower extremity (2.32% vs. 2.42%). In our study, both the control and experimental groups displayed a similar distribution of TBSA, indicating that the baseline characteristics of the patients were comparable. Among the participants, nine patients had a TBSA of less than 10%, seven between 10% and 20%, and four had a TBSA greater than 20%. Notably, within the group with a TBSA above 20%, the control group included one patient with a TBSA of 31%, while the experimental group had two patients with TBSA measurements of 30% and 30.5%. These findings are significant for assessing the effectiveness of the experimental treatment in comparison to the standard of care, as TBSA serves as a vital indicator of wound severity and informs treatment planning.

**Figure 4:**
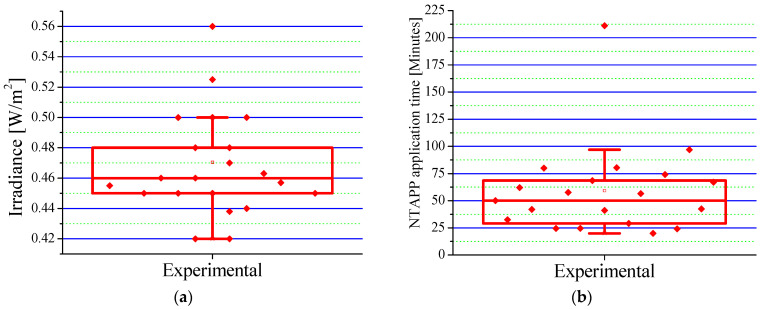
Parameters of the NTAPP in the experimental group: (**a**) distribution of irradiance applied to patients; (**b**) distribution of NTAPP application time per patient. The blank square represents the mean value. In (**a**), the diamonds represent the irradiance for each patient, and in (**b**), the application time for each patient.

Patients in the experimental group received an average irradiance of 0.4659 ± 0.054 W/cm^2^ (range: 0.420–0.560 W/cm^2^), as shown in Figure 4a. This irradiance, significantly lower than the safety limit established by the ICNIRP [33], ensures the safety of the treatment and facilitates tissue repair by modulating inflammatory processes. The results indicate that combining Epifast^®^ and NTAPP at this dose is a safe and effective therapy to optimize the healing process and significantly improve a patient’s quality of life.

The mean fluence used during NTAPP application was 0.7677 °C/cm^2^, complying with internationally established safety parameters. The analysis of treatment duration (Figure 4b) revealed an average total time of 59.20 min per patient, distributed over an average of five sessions. Each session, with an average duration of 11.84 min, proved not only manageable but also highly efficient, instilling confidence in the protocol’s effectiveness. Considering an average of TBSA burned of 12.55% (see Table 2), approximately 1.06 min was required for each 1% of TBSA. These results are consistent with previous research [5,16], validating the efficacy and reproducibility of our protocol. Variability in treatment time can be attributed to factors such as burn depth, extent, and patient age.

The analysis of burn unit arrival times revealed a slightly higher average in the experimental group (21.35 h) than in the control group (17.06 h). However, this difference was not statistically significant (*p* = 0.69). Conversely, the experimental group showed less variability in arrival times. The relationship between burn extent and arrival time was similar in both groups: patients with less than 10% of TBSA had the most extended arrival times, while those with more than 20% of TBSA had the shortest arrival times. Despite these general trends, no significant interaction was found between the study group and burn extent, suggesting that the influence of burn extent on arrival time was similar in both groups. These results indicate that, although there are some subtle differences in arrival time, access to specialized care was comparable between the control and experimental groups.

Our findings reveal a clinically significant difference in outcomes between the treatment groups. Despite having similar burn extents, the experimental group required significantly fewer skin grafts (2 ± 0.00 vs. 8 ± 0.93 in the control group; *p* = 0.02). This substantial reduction translates into a reduced need for skin grafts, with only 10% of patients in the experimental group requiring this procedure, compared to 40% in the control group. These results suggest that the experimental protocol promoted faster and more complete healing, significantly decreasing the reliance on additional surgical procedures. Reducing the need for grafts minimizes the risks of associated complications, such as rejection and infections, resulting in lower postoperative morbidity.

The analysis revealed that the experimental group (meaning 3.30 surgeries, standard deviation (SD) = 1.5593) required slightly fewer surgical procedures than the control group (4.35 surgeries, SD = 3.2650). However, this difference was not statistically significant (*p* = 0.56), suggesting that variability in the data could mask a possible treatment effect. Despite this lack of statistical significance, the trend toward fewer surgeries in the experimental group is important.

When specifically analyzing the length of hospital stay associated with the complete epithelialization process (Table 3), a clinically relevant trend is observed: the control group had an average of 11.65 days of hospitalization, while in the experimental group, this value was reduced to 9.85 days (*p* = 0.38). Although this difference did not reach statistical significance, the consistent decrease in hospitalization time related to wound healing in the experimental group suggests a potential clinical benefit of the treatment. The analysis of hospital discharge rates reinforces this trend. On the sixth day post-treatment, 40% of patients in the experimental group had already been discharged, compared to 20% in the control group. On day 14, this difference widened even further, with 80% of discharges in the experimental group versus 75% in the control group. These findings indicate that experimental treatment could promote the healing process, allowing for faster recovery and significantly reducing hospital stays.

Regarding the analysis of hospital discharges per day, the control group recorded four patients (20%) discharged on the sixth day. In comparison, the experimental group had eight patients (40%) discharged on the same date. By day 14, fifteen patients in the control group (75%) and sixteen in the experimental group (80%) were discharged.

This study evaluated analgesic use, the incidence of skin infections, and antibiotic use as critical indicators in burn patient management. The control group showed higher analgesic consumption (21.15 doses vs. 13.01 doses in the experimental group), though not statistically significant (*p* = 0.09), indicating potential differences in pain management strategies.

When analgesic consumption was assessed in burn patients, considerable variability was observed between the study groups. Although no significant differences were found in total analgesic consumption between the control and experimental groups (*p* = 0.09), the analysis revealed a trend toward higher consumption in patients with a higher percentage of TBSA. These results suggest that while TBSA may influence the need for analgesia, it is not the only factor. Other factors, such as burn depth, complications, patient age, and individual analgesic responses, also play a significant role. This approach, which optimizes pain control, has the potential to significantly improve the quality of life of burn patients.

The incidence rate of healthcare-associated infections was notably lower in the experimental group (25%) than in the control group (37.95%), a significant finding. This reduction underscores the effectiveness of the interventions. Similarly, the average antibiotic consumption was lower in the experimental group (5 doses, SD = 7.52) compared to the control group (7.95, SD = 12.92). Pearson correlation analysis revealed a weak positive association between the infection rate and antibiotic consumption (*r* = 0.206, *p* = 0.38). However, this relationship was not statistically significant. Although a trend toward higher antibiotic consumption was observed in patients with a higher infection rate, other factors, such as disease severity, comorbidities, and variability in individual responses to antibiotics, could have masked this relationship. For instance, patients with a more severe disease might require more antibiotics, regardless of their infection rate.

These findings underscore the potential impact of the interventions implemented in the experimental group on infection control. The successful reduction in nosocomial infections and antibiotic consumption, a significant step forward, instills optimism and hope for improved patient care. The experimental group’s lower variability in antibiotic consumption suggests greater homogeneity in antibiotic management. However, it is essential to note that the relationship between the infection rate and antibiotic consumption is complex and multifactorial.

This study also assessed the impact of NTAPP as an adjuvant therapy on blood product consumption. Data analysis showed that both groups received comparable transfusions of packed red blood cells and blood plasma. However, statistical analysis revealed a significant difference in the platelet concentration requirement: the experimental group required no units, whereas the control group required an average of seven units. This marked difference suggests that NTAPP may have benefitted primary hemostasis, consistent with its known ability to stimulate growth factor production and modulate the inflammatory response. These preliminary results strongly support the hypothesis that NTAPP therapy has excellent potential to reduce the need for platelet concentrate transfusions and improve clinical outcomes in patients.

The following supplemental data analysis provides additional evidence supporting the efficacy of experimental NTAPP treatment in managing pediatric second-degree burns. The data indicate that scald burns were the most common cause of injury, accounting for 72.5% of cases, followed by fire-related burns at 20% and electrical burns at 7.5%. Notably, 80% of incidents occurred between midday and midnight, highlighting the need for preventive strategies during this critical period. In terms of the TBSA affected by burns, the control group averaged 12.60%, while the experimental group showed a similar average of 12.55%. However, regional variations in TBSA may have influenced the treatment response. Clinically, a significant difference was observed in the reduced need for skin grafts in the experimental group, which required an average of two grafts compared to eight in the control group (*p* = 0.02). This reduction implies less surgical morbidity and indicates more effective healing and faster recovery. Although there was no significant difference in the total number of surgeries (control: 4.35, experimental: 3.30, *p* = 0.56), the trend toward fewer surgeries in the experimental group suggests a positive effect of the treatment. Additionally, a trend toward shorter hospital stays was noted in the experimental group, averaging 9.85 days compared to 11.65 days in the control group (*p* = 0.38), which may lead to cost savings and improved hospital efficiency. Pain management also showed notable differences, with the experimental group consuming fewer analgesics (13.01 doses) compared to the control group (21.15 doses, *p* = 0.09). Moreover, the experimental group exhibited a significantly lower infection rate of 25% compared to 37.95% in the control group, suggesting that NTAPP treatment provides a protective effect against secondary infections. Finally, the experimental group did not require platelet concentrates, while the control group averaged seven units. This finding implies an improvement in primary hemostasis. Overall, these data support the hypothesis that NTAPP treatment reduces the need for invasive surgical interventions and enhances clinical outcomes in terms of infection prevention, pain management, and overall patient recovery.

**Figure 5:**
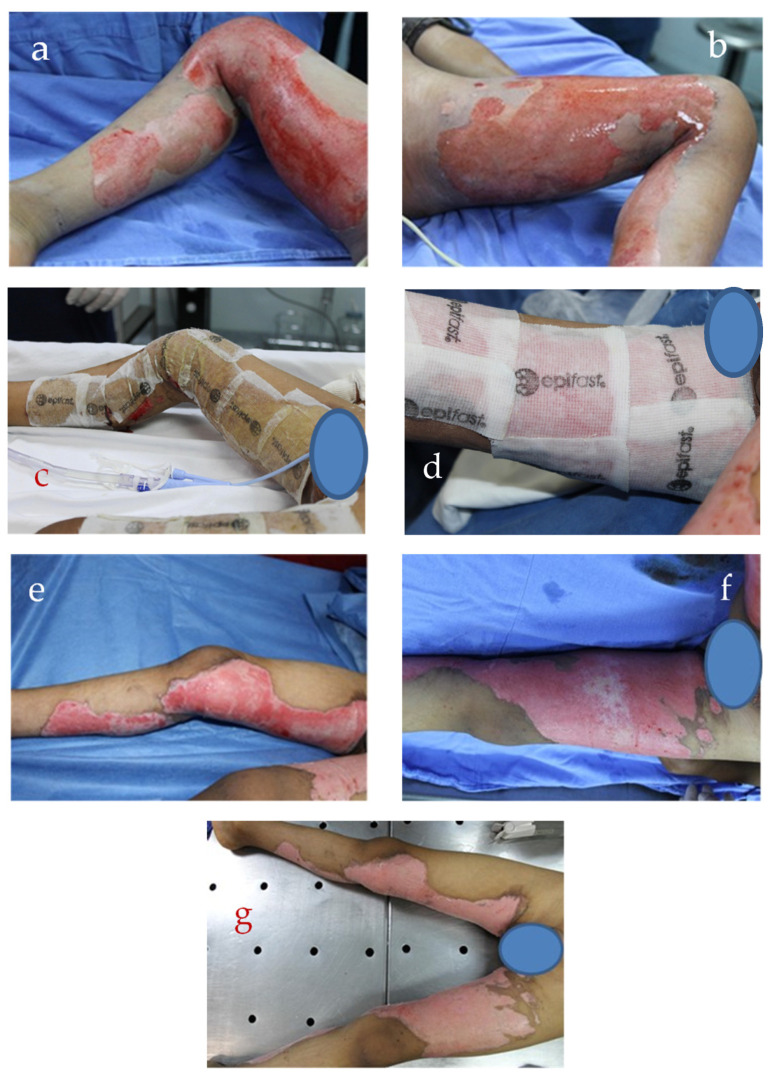
Clinical evolution of the patient after treatment with NTAPP for superficial partial-thickness burns. (**a**,**b**) show the state of the wound immediately after cleaning and applying the NTAPP treatment; (**c**,**d**) show the application of Epifast^®^ to the affected regions; (**e**,**f**) illustrate the evolution of the tissue three days after treatment, showing an improvement in the appearance and characteristics of the wound; (**g**) presents the result ten days after treatment, where a notable tissue regeneration and a significant reduction in inflammation can be observed.

Figure 5 illustrates the clinical course of a patient presenting with a second-degree burn. In Figure 5a,b, the initial assessment reveals a profoundly red, blistered second-degree burn following cleansing. Epifast^®^ was applied to the affected regions (Figure 5c,d). A marked improvement was observed after just five days of NTAPP treatment (Figure 5e,f). Re-epithelialization concurrently diminishes, and redness and inflammation visibly diminish. Complete wound re-epithelialization is achieved by day 10 (Figure 5g).

## 4. Discussion

In this study, we investigated the potential of NTAPP, generated by the reactor described in Section 2.2, as an adjuvant treatment for pediatric patients with superficial partial-thickness and deep dermal burns. The age distribution between the experimental and control groups was comparable, with mean ages of 4.14 years and 4.20 years, respectively. This similarity in age distribution ensures a balanced comparison and minimizes the potential confounding effects on the study outcomes [39]. Furthermore, our findings corroborate the established prevalence of scald burns, particularly in children aged 1 to 4 years. Specifically, scald burns accounted for 65% of cases in the experimental group and 50% in the control group. This observation aligns with the existing literature, which consistently identifies scalds as the predominant cause of pediatric burns [40]. Consequently, these data underscore the critical role of scald burns as a leading cause of admission to pediatric burn units [41].

The temporal patterns of burn injuries in pediatric patients reveal a significant prevalence during the high-risk period of 12 to 24 h. This finding aligns with previous studies in both the control and experimental groups [42]. Specifically, in the control group, 30% of burns occurred between 12 and 18 h, with scalds being the primary cause, followed by 20% due to fire-related incidents. During the 18 to 24 h period, scalds accounted for 40% of burns, while fire and electrical injuries each comprised 5%. In the experimental group, scalds represented 20% of burns between 6 and 12 h, another 20% between 12 and 18 h, and an additional 35% between 18 and 24 h. Fire and electrical injuries each accounted for 10% of burns between 12 and 18 h and 5% between 18 and 24 h. These results are consistent with prior publications [42,43,44], validating the reliability and relevance of our data regarding the temporal distribution of pediatric burn injuries.

The distribution of patients across TBSA categories (<10%, 10–20%, and >20%) showed comparable proportions between the experimental and control groups. Notably, patients with more extensive burns tended to present to the burn unit earlier, indicating a correlation between the burn severity and time of presentation. This trend was observed in both groups; however, patients in the experimental group with severe burns presented significantly earlier than those in the control group. These findings are consistent with prior research, which has also reported a correlation between burn severity and expedited presentation to medical facilities [45,46,47].

Prior research underscores the significant positive impact of the experimental treatment in reducing the need for skin grafts compared to standard hospital practices. Despite comparable TBSA between groups, the marked difference in graft utilization highlights the superior clinical efficacy and therapeutic benefits of the experimental approach. This substantial reduction in graft requirements within the experimental group suggests an accelerated healing process, potentially leading to shorter hospitalizations and decreased healthcare costs. The integration of NTAPP as an adjuvant therapy demonstrates considerable potential for enhancing patient outcomes, especially in individuals with second-degree burns. Ongoing studies by various researchers employing alternative methodologies [48,49] further validate the critical role of innovative treatments in burn management.

The rate of epithelialization in pediatric patients with second-degree burns, regardless of depth, is a critical determinant of the healing process. Consistent with prior studies [50,51,52], our findings demonstrate that the extent of the burned surface significantly influences epithelialization time. Patients with smaller burn areas in both groups exhibited accelerated epithelialization. Notably, those treated with a biological dressing of human keratinocyte cultures (Epifast^®^) in conjunction with NTAPP experienced a 15.5% reduction in epithelialization time, correlating with shorter hospital stays.

These findings underscore the importance of individualized patient assessment in burn management to optimize epithelialization rates and enhance long-term functional outcomes. While NTAPP demonstrates significant adjuvant benefits, its integration within a comprehensive, patient-specific therapeutic strategy is essential for maximizing recovery in pediatric burn patients.

Importantly, none of the patients in the experimental group reported discomfort during NTAPP administration [53,54]. This absence of reported discomfort is a significant clinical finding, as it directly impacts patient care and recovery. Furthermore, while general anesthesia was required for the initial surgical intervention in the experimental group, subsequent NTAPP applications were performed without the need for sedation or analgesia, and no adverse sensations were reported. These observations further substantiate the safety profile and clinical utility of NTAPP as a feasible treatment modality for burn patients.

Secondary bacteremia in burn patients presents a significant clinical challenge due to infection risk. We implemented a systematic antibiotic prophylaxis strategy using broad-spectrum antibiotics (dicloxacillin and aztreonam) preoperatively and as needed. Initial empiric therapy included vancomycin and ciprofloxacin, with regimens adjusted based on culture-confirmed bacterial susceptibility to minimize antibiotic overuse. However, conventional prophylaxis may not consistently reduce infection-associated morbidity. Our experimental group showed a non-significant trend toward decreased antibiotic use. NTAPP treatment, with its antiseptic properties and ability to reduce microbial bioburden, may improve outcomes and complements our antibiotic strategy. We believe NTAPP integration with surgical interventions like debridement can enhance clinical results and reduce postoperative morbidity [55,56,57].

Finally, we observed that 40% of patients in the control group required grafting, compared to only 10% in the experimental group, a statistically significant difference (*p* < 0.02). These results unequivocally demonstrate that NTAPP therapy has the potential to be a highly effective adjuvant in the treatment of both superficial partial-thickness and deep dermal second-degree burns. Therefore, it is imperative to investigate how debridement before NTAPP application influences patient outcomes and wound healing.

This study shows promising results for using NTAPP in treating pediatric burns; however, it is important to acknowledge its limitations. Consequently, large-scale multicenter studies that involve a diverse patient population are necessary. Additionally, it is vital to investigate how NTAPP affects different types of burns and patients with specific comorbidities. Examining the molecular mechanisms behind its antimicrobial and anti-inflammatory properties, as well as directly comparing it to other adjuvant treatment methods, are crucial steps in establishing evidence-based administration protocols. Finally, long-term follow-up studies are needed to evaluate the impact of NTAPP on patients’ quality of life and overall recovery.

## 5. Conclusions

This study demonstrates the promising potential of non-thermal atmospheric pressure plasma (NTAPP) as an adjuvant in the treatment of superficial partial-thickness and deep dermal burns in children. The significant reduction in the need for skin grafts, a central finding, validates the efficacy of NTAPP and suggests an advancement in the management of pediatric burns. By minimizing surgical intervention, especially when combining NTAPP with Epifast^®^, tissue repair is optimized, and a trend toward reduced hospitalization time is observed, directly translating into a substantial improvement in patients’ quality of life. Although the consumption of analgesics and antibiotics showed no statistically significant differences, the marked decrease in surgical morbidity suggests a reduction in pain and local inflammation, an effect attributable to the antimicrobial and anti-inflammatory properties of NTAPP. Taken together, these results strongly support the integration of NTAPP as a key adjuvant therapy in the treatment of pediatric burns. The synergy between NTAPP and biological dressings such as Epifast^®^ represents an innovative strategy to accelerate recovery and improve clinical outcomes. By opening new avenues for less invasive and more effective treatments, this study not only improves burn management but also advances research into advanced regenerative therapies, with the potential to revolutionize clinical practice in the field of regenerative medicine.

## Figures and Tables

**Table 1:** Distribution of patients by age and sex.

Age [Years]	Female Patients		Male Patients		Total Patients		Percentages of Female and Male in Control and Experimental Groups [%]
	Control Group	Experimental Group	Control Group	Experimental Group	Control Group	Experimental Group	
0–1	0	1	0	1	0	2	0/5.0
>1–4	6	6	7	6	13	12	32.5/30.0
>4–9	3	1	2	2	5	3	12.5/7.5
>9–14	0	0	2	1	2	1	5.0/2.5
>14	0	1	0	1	0	2	0.0/5.0
Total patients	9	9	11	11	20	20	50.0/50.0
Average age	3.23	4.57	5.18	4.69			

**Table 2:** Comparison of results and the use of grafts between the standard treatment protocol and NTAPP.

Diagnosis	Control Group	Experimental Group	*p*
%TBSA	12.60 ± 8.2	12.55 ± 8.06	0.98
TBSA < 10	6.33 ± 1.67	5.88 ± 1.57	
TBSA 10–20	13.75 ± 3.91	13.88 ± 3.85	
TBSA > 20	28.33 ± 4.61	27.83 ± 4.19	
Average time between the burn and admission to the hospital [hours]	17.06 ± 35.79	21.25 ± 27.14	0.69
TBSA < 10 [hours]	37.45 ± 50.91	36.65 ± 38.53	
TBSA 10–20 [hours]	15.18 ± 8.81	11.53 ± 6.45	
TBSA > 20 [hours]	8.84 ± 7.24	9.30 ± 7.24	
Used grafts number	8 ± 0.00	2 ± 0.00	0.02
TBSA < 10	0.33 ± 0.00	0.12 ± 0.00	
TBSA 10–20	0.37 ± 0.00	0.11 ± 0.00	
TBSA > 20	0.66 ± 0.00	0.00 ± 0.00	

**Table 3:** Analgesic, antibiotic usage, and skin infection incidence in burns: control group vs. experimental group.

Group	Analgesic [Doses]	Incidence of Skin Infections [%]	Antibiotic Use [Mean Doses]	Antibiotic Use	Hospital Stay [Days]
Control	21.15	25	7.95	Higher in 10% of TBSA	11.65
Experimental	13.01	10	5.00	Higher in 10–20% of TBSA	9.85

## Data Availability

The datasets featured in this article are not currently accessible as they are securely stored within the Pediatric Burn Unit at Dr. Nicolás San Juan Hospital and are part of individual patient records.
